# Academic promotion packages: crafting connotative frames

**DOI:** 10.1007/s40037-016-0304-2

**Published:** 2016-10-20

**Authors:** Lara Varpio, Christina St.Onge, Meredith Young

**Affiliations:** 1 Uniformed Services University of the Health Sciences, Bethesda, MD USA; 2 Université de Sherbrooke, Sherbrooke, Québec Canada; 3 McGill University, Montreal, Québec Canada

**Keywords:** Teaching, Scholarship, Tenure and promotion, Health professions education

## Abstract

Among the challenges of navigating the promotion and tenure (P&T) process is the need to describe one’s career using the language of P&T expectations, while also framing that language to reflect the unique work involved in health professions education (HPE) scholarship. Drawing on the distinction between denotative and connotative meanings of words, we describe how the language of P&T standards can hold different meanings depending on how they are contextualized in the HPE field and the communities therein. To illustrate, we describe our experiences of adapting the language of ‘teaching’ to the expectations of the P&T committee while also reflecting the non-traditional ‘teaching’ we do in HPE. We also share three practical tips for navigating the P&T process: (1) find a local mentor, (2) craft the story of your expertise, and (3) seek feedback from your local stakeholders on the connotative story you have crafted.

## Introduction

Every few years, some version of this joke is shared around academic circles:

Why didn’t Socrates get promoted?

- He didn’t have any publications. Not one.
- He collected no teaching evaluations so there was no evidence that his teaching resulted in student learning (i. e., students may have learned the course content from Plato, the smartest student in class).

This joke gets laughs because it is ridiculous – any academic community (from Departments of Philosophy to Internal Medicine) would benefit immeasurably from having someone of Socrates’s intellectual calibre on staff. But could he pass muster with the Promotions and Tenure (P&T) committee?

This question haunts many clinician- and PhD-trained faculty members. Few of us can rely on Socratic levels of intellect to impress the P&T committee. And so we pour over P&T guidelines. We spend hours devising tactics with our mentors. We consider how each email received during the course of our careers might testify to different academic skills.

Like with so many other aspects of our professional careers, we approached the challenge of creating a P&T package as a scholarly endeavour. We researched the definitions of and expectations behind each P&T requirement. We set out to collect evidence to demonstrate achievements that would prove us worthy of promotion. Eventually, we realized that our scientific approach was only a partial solution to the challenge. We realized that crafting a persuasive P&T package was both a science and an art.

In this paper, we share the lessons we learned associated with the artistry of compiling a convincing P&T package. Specifically, we highlight how P&T terms hold different meanings depending on how they are contextualized by specific academic audiences (in our context, by some of the different audiences of health professions education [HPE]). We illustrate these differences by describing how the term ‘teaching’ has variable meanings to different HPE audiences. Further, we share the frame we have crafted for our P&T submissions, and provide some practical tips that supported our tenure- and/or promotion-chase efforts.

## Denotative vs connotative meanings

Each word has both a denotative and a connotative meaning. A word’s denotative meaning is the objective, literal definition that one would find in the dictionary. A word’s connotative meaning is the culturally specific, individual, and emotional associations that nuance a word’s meaning. To illustrate, consider a word that has powerfully different denotative and connotative meanings: feminist*. *A feminist is denotatively defined as a person who believes that ‘men and women should have equal rights and opportunities’ [1]. This dictionary-based meaning acquires different social and personal overtones in different contexts (e. g., being called a *feminist* at a Beyoncé concert is very different than being a *feminist* research scholar).

Constructing a promotion package requires presenting your academic career using the denotative terms set out by the P&T requirements, but framing those terms with connotative meanings that reflect HPE’s cultural and academic contexts. In other words, our P&T packages need to respect the denotative meanings of ‘teaching’, ‘research’, ‘service’ (i. e., the academic trifecta grounding many P&T considerations) while simultaneously framing academic accomplishments in these areas with HPE’s connotative associations. Our original scientific approach to crafting our P&T packages had us focusing on the denotative meanings of the trifecta terms.

To illustrate, we spent considerable time reflecting on the ‘teaching’ activities we could include in our P&T packages. We built a tool with which to collect evaluative data on our teaching skills in order to support that element of our P&T packages. We constructed that tool to meet the denotative meaning of post-secondary teaching. We quickly learned, however, that the denotative meaning of post-secondary teaching holds variable connotative meanings for different HPE audiences.

We determined that much of the teaching work we did was through one-to-one collaborations with individuals (primarily clinicians) interested in engaging in HPE scholarship. In these dyads, we typically work with clinicians to build high-quality scholarly investigations within the HPE domain. We share our expertise in securing ethics approval, employing different methods and methodologies for data collection and analysis, and disseminating findings to the broader HPE community.

While this work is congruent with our understanding of the term ‘teaching’, we soon realized that others did not necessarily share the same associations. For instance, PhD-trained HPE research scientists were uncomfortable universally labelling this work as teaching for two main reasons. First, they were concerned that the clinicians they worked with would not recognize that one-to-one collaborative work as teaching. They felt the clinicians might reject framing themselves as learners in those conversations. Second, they wanted to protect the collaborative relationships they had built with their clinician colleagues. While they described having different ‘lessons’ ready to help clinicians get to the next stage of the research process, they did not like the ‘teaching’ label. They worried it would change the qualities of their collaborations, that those collaborations would become inappropriately formal.

We then considered labelling that work as mentoring, a term that the P&T regulations would acknowledge as teaching, but that might avoid the connotative problems described by our PhD-trained colleagues. We were hopeful that this label would be well received since the teaching activities we were describing have been identified as the work of a ‘scholarly mentor’ in the medical education literature [2]. Unfortunately, that framing brought its own challenges. We learned from members of different medical schools’ leadership that we could not have official ‘mentor’ status. We learned that, in many contexts, mentors have to hold a senior rank at the university or medical school (i. e., be at the associate or full professor rank). So, from an organizational point of view, we were not eligible to describe our teaching as mentoring because we had not, at that time, been promoted.

Next, we considered drawing on the language of clinical consults. We hoped this would bring to mind connotations of ‘teaching’ that would be acceptable to P&T committees and our HPE community members. Again, administrative leaders told us that consulting work would not qualify as teaching for promotional purposes. From their perspective, consultants stand external to the organization and get paid for specific tasks. Therefore, consulting is not part of a faculty member’s teaching work. Furthermore, our PhD-trained colleagues did not want to be labelled consultants because that held connotations of service-oriented roles. They were concerned that being labelled as a consultant would minimize their intellectual contributions to the scholarly work. Furthermore, they were concerned that this label would undermine the collaborative relationships that they nurtured with their clinician collaborators.

**Fig. 1:**
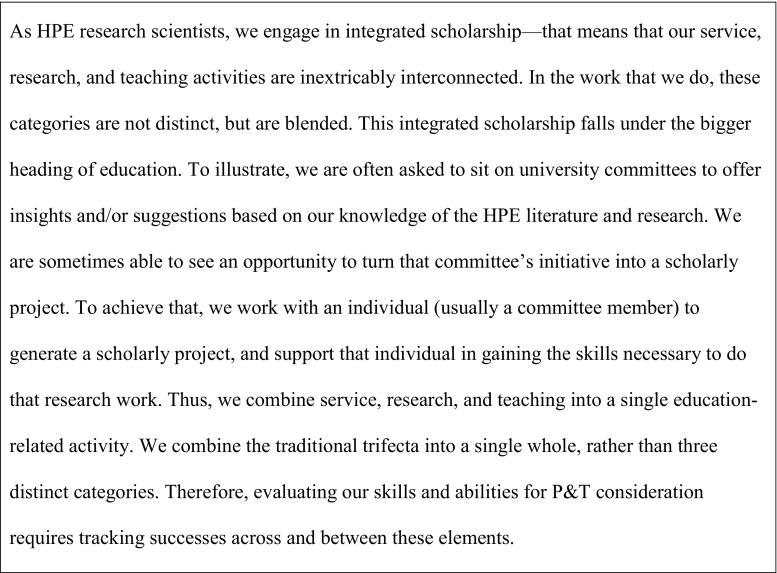
How we connotatively frame our P&T packages

As this example illustrates, reporting our ‘teaching’ activities to the P&T committee was challenging because of the term’s connotative variability in different contexts and even with different audiences in a single context. We should note that we also had similar challenges with the terms ‘research’ (e. g., given the limited availability of HPE-related grants [3], how much grant money needs to be secured for an HPE researcher to be deemed successful?) and ‘service’ (e. g., if we see and act on an opportunity for scholarship based on participation in, for example, a curriculum evaluation committee, does that work count towards ‘service’ or ‘research’ activity?). In the end, we realized that promotion packages are challenging to construct precisely because the denotative meanings of the P&T requirements are not consistently congruent with the connotative meanings held by members of the HPE community. However, we realized that this incongruity offers the solution to the challenge. When we let go of strict, denotative meanings, we discovered the freedom afforded to us via connotative framings. For us, that meant re-framing our teaching, research, and service work in completely different ways (see Fig. 1). Once we abandoned the idea that teaching, service, and research were three separate categories, we were able to reimagine a connotative frame that more aptly described the nature of our HPE careers.

Interestingly, research into the expectations of academics in higher education has increasingly reported the need for professors to integrate their teaching and research activities [4–7]. Scholars investigating the integration of teaching and research have cautioned that contextual factors are fundamental in shaping the nature of this integration [8]. This research cites national, disciplinary, and local political contextual factors as having a crucial role in shaping the teaching-research nexus [8]. We suggest that these contextual factors also shape the connotative meanings of academic productivity and impact measures, especially the measures of research, teaching, and service work. We note that research into this integration has yet to include service activities in the nexus. We posit that in HPE (a) the integration should be expanded to include research, teaching, and service activities, and (b) this integration needs to shape (both denotatively and connotatively) the P&T expectations of faculty members.

## Tips for navigating the denotative/connotative divide

Promotional processes are different at each institution. These idiosyncrasies mean that there is not a single, transferable approach for crafting a persuasive HPE promotion package. The frame we constructed to contextualize our promotion package materials may not work for others. Thus, instead of offering tips for what to write or which documents to include in a P&T package, we offer the following three suggestions for how to approach the process of crafting the promotion package:

1. A local mentor is an absolute *must-have *collaborator. Finding a mentor who knows and understands the local P&T processes and committee is essential for translating your academic work to the P&T committee. As our experience with ‘teaching’ illustrates, P&T categories can be perceived in different ways by different audiences. A local mentor will know which connotative frame will resonate with your committee, and will have strategies for deflecting the objections of readers.
2. Do not treat promotion criteria as merely objective, denotative definitions. While it is important to understand the denotative meanings of each criterion, it is equally important to find the connotative associations that fit your HPE activities *and* that resonate with the members of the P&T committee. Work to develop the ‘story’ of your work that reflects your connotative meanings *and* respects the denotative requirements imposed by the P&T process. Craft your package to be congruent with both connotative and denotative definitions.
3. Part of constructing the promotion package is testing out the connotative frame with your local community and gathering evidence that fits the frame. We asked the clinicians we worked with to complete an evaluation form so we could demonstrate our skill and effectiveness in the educational work we do. In so doing, we tested our connotative frame. These clinician collaborators helped us to refine our narrative and to understand the reactions of different audiences. Those conversations provided invaluable feedback on our connotative frame, and the evidence we can use to support it.

## Conclusion

In crafting our P&T packages, we have gained an appreciation of the institutional specificity of P&T processes, and of the connotative associations of the terms we have used in our HPE work. Socrates would surely get promoted today, but he too would have to learn to describe his work in ways that create connotative frames to nuance and contextualize the P&T committee’s denotative expectations. The fact that he did not publish a single work could probably be defended. We imagine, however, that his students might request a Podcast version of his lessons.
